# A study of auditory evoked potentials in systemic lupus erythematosus patients

**DOI:** 10.1016/S1808-8694(15)30579-6

**Published:** 2015-10-19

**Authors:** Maíra dos Santos da Mata Rezende, Maria Cecília Martinelli Iório

**Affiliations:** 1 Master's degree student in Human Communication Disorders; speech therapist.; 2 Livre-Docente (habilitation) professor in Human Communication Disorders. Adjunct professor of the Hearing Loss Discipline, Speech Therapy Department, Universidade Federal de São Paulo - Paulista School of Medicine (EPM). Universidade Federal de São Paulo - Paulista School of Medicine (EPM).

**Keywords:** systemic lupus erythematosus, auditory evoked potentials, auditory brain stem evoked potentials, event-related potentials p300

## Abstract

Systemic lupus erythematosus (SLE) is a multifactorial chronic systemic inflammatory disease, of unknown origin, characterized by the presence of autoantibodies and polymorphic clinical manifestations. This disease may involve multiple organs and systems. The most common findings are articular, cutaneous, vascular, renal, neurological, cardiac, gastrointestinal, hematological, ocular, and auditory abnormalities.

**Aim:**

To investigate the central auditory function of subjects diagnosed with Systemic Lupus Erythematosus (SLE).

**Material and Method:**

A time-series study was made of sixty subjects, aged between 21 and 46 years, which were divided into a control (n=30) and an experimental group (n=30). A clinical history, audiological evaluation (pure tone audiometry, speech audiometry and immitance testing) and short (ABR), middle (MLR) and long latency (LLAEP) potentials were carried out in all subjects. Results: No statistically significant differences were observed between the groups in any of the evaluations.

**Conclusions:**

In this study there were no differences in short, middle and long latency auditory potentials between the control and experiment subject groups..

## INTRODUCTION

Systemic lupus erythematosus (SLE) is a multifaceted chronic systemic inflammatory disease of unknown origin that affects predominantly young women, and which progresses with periods of illness alternating with remission. It may be characterized immunologically by the presence of autoantibodies; its clinical manifestations are varied. This disease may affect multiple body organs and systems, the most common being joints, the skin, the vascular system, kidneys, the neurological system, the heart, the gastrointestinal system, the hematological system, the eyes and the auditory system.^1^

Primary central nervous system (CNS) involvement is common in SLE patients; thus, any area of the brain, the spine or the nervous system may be affected. Some of the symptoms are cognitive alterations, seizures, psychosis and headaches.^2^

To be more precise, in some cases short and long term memory processing and verbal and visual-spatial information processing are affected. Attention may also be significantly compromised, especially in SLE that present with neuropsychiatric manifestations.^3^

SLE patients frequently report peripheral auditory alterations (sensorineural hearing loss).^4^, ^5^, ^6^ However, there have been few reports in the literature about central auditory alterations in SLE patients.

Normal hearing requires whole auditory system structures operating adequately from the external ear to the auditory cortex.

Anatomical and physiological wholeness of the peripheral and central auditory system is essential for language and speech acquisition and development. Thus, an association of objective and subjective behavioral methods for auditory evaluation has become increasingly frequent in audiology to support and add precision for the diagnosis of central and/or cognitive auditory disorders.

Pure tone audiometry is the most frequently used test in audiological diagnosis. This test, however, investigates only peripheral hearing by obtaining air and bone auditory thresholds.

Electrophysiological tests, also named auditory evoked potentials (AEPs), have been used for assessing central audition. These tests evaluate the neuroelectrical activity of the auditory pathway from the auditory nerve to the cerebral cortex in response to an acoustic stimulus or event. These potentials, which are generated by sequential and synchronous activation of nervous fibers along the auditory pathway,^8^ may be picked up by surface electrodes placed on various sites of the head.^7^

AEPs reveal the integrity and functional ability of the central auditory nervous system and may confirm the injury site.^9^ AEPs may be classified according to their latency (time between the presentation of the stimulus and the response) as short, middle, and long-latency potentials.

Short-latency brainstem auditory evoked responses, also known as brainstem auditory evoked potentials (BAEP), are used more frequently in the clinical setting due to their reproducibility and locating properties. They occur from zero to 10 milliseconds (ms) following the presentation of an acoustic stimulus.^10^ These potentials assess the integrity of auditory pathways in the brainstem, and may be used to infer data about peripheral auditory sensitivity. A set of seven waves generated by one or more structures along the auditory pathway may be seen in response to acoustic stimulation.

Möller et al.^11^ described the following BAEP wave generating sites: Wave I - distal portion to the brainstem of the auditory nerve; Wave II - proximal portion to the brainstem of the auditory nerve; Wave III - cochlear nucleus; Wave IV - superior olivary complex; Wave V - lateral lemniscus; Wave VI - inferior colliculus; Wave VII - medial geniculate body.

The following parameters are used for analyzing the responses: absolute latencies of waves I, III, V and interpeak latencies I-III, III-V and I-V relative to the stimulus intensity; also used are the response amplitude, morphology and reproducibility.^12^

Mid-latency auditory evoked potentials (MLAEP) have no specific generating site; they reflect global cortical and subcortical activation. Estimates suggest that their most important response generators are located in the thalamus and the primary auditory cortex.^13^ MLAEP occur between 10 and 100ms following an acoustic stimulus, arising soon after brainstem potentials.^14^

The P300 or P3 are the most commonly used long-latency auditory evoked potentials (LLAEP) in clinical practice; they are used for investigating cognition and attention. These potentials are obtained by focusing attention on a rare stimulus. It occurs about 300ms after stimulus presentation and is generated by frequent stimuli among which a different (rare) stimulus occasionally occurs. The different stimulus appears 15 to 20% of times, and subjects are asked to normally identify the rare stimulus, mentally counting how many times it occurs. The auditory systems becomes used to hearing a frequent stimulus as fewer neurons respond to it. Rare stimuli, on the other hand, are heard few times; the system, therefore, responds to these with more neurons. The curve these neurons generate is larger than that generated by the frequent stimulus. Subtracting both stimuli yields the P300.^15^

It is a consensus in the literature that the P300 has multiple generators. The main generators, according to Mc Pherson,^16^ are the frontal cortex, the centroparietal cortex and the hippocampus.

The purpose of this study was to investigate central auditory function in SLE individuals.

## MATERIAL AND METHOD

The Research Ethics Committee of the institution in which the study was conducted approved its design (protocol nº. 0221/05).

This was a case control study. The sample consisted of 60 female individuals, of which 30 became the study group and 30 became the control group. Both groups were age-paired. The age ranged from 21 to 46 years.

Inclusion criteria for the study group were the following: SLE subjects according to the definition of the American Rheumatology Association (1982);^17^ age between 13 and 46 years to avoid the effect of CNS aging on the evaluation; no clinical history of risk factors for hearing loss as described by the Joint Committee on Infant Hearing;18 no external or middle ear involvement of any sort; no past use of hearing aids.

Inclusion criteria for the control group were the following: good health; no peripheral and/or central auditory involvement of any sort; no neurological and/or cognitive alterations; no evidence of syndromic diseases.

Subjects for the study group were selected through a survey of charts when patients visited the rheumatology outpatient unit. These individuals were assessed on the same day in which they visited the rheumatology outpatient unit or at a near date of their choice. Subjects for the control group were selected from patients in the waiting room of the audiology outpatient unit.

## PROCEDURES

After reading and signing the free informed consent form, subjects were interviewed for their clinical history and then underwent testing of AEPs.

Subjects received orientation for testing of AEPs as follows: avoid taking tea, coffee and chocolate in the preceding 24 hours; avoid intense physical or mental activity on the day before testing; avoid smoking and drinking alcoholic beverages preferably 24 hours before testing; avoid using hair gel and face creams before testing.

Testing of AEPs was done using the Bio-logic® systems corp. software with four channels, in an electrically protected and acoustically isolated ambience. Skin was cleansed with gauze and abrasive paste. Surface electrodes were then placed over electrolytic paste (for optimizing electrical conductivity) and fixed with microporous adhesive tape.

First, the long-latency AEP (P300) was measured, followed by the MLAEP and the BAEP. This sequence was chosen because the generation of P300 and MLAEP is influenced by alertness and attention to the sound stimulus, which BAEPs may be generated in the waking state and during sleep.

Electrode impedance values were set below 5 kOhms (k). Patients were asked to lie down and remain as relaxed as possible. Testing was done in a darkened room at a pleasant temperature. Artifacts were controlled during AEP testing (≤ 10%) so as not to interfere on the stimulus and response recording.

For BAEP recording, electrodes were placed on the brow (Fpz = ground electrode), on the cranial vertex (Cz = active electrode), ear lobules (A1 = left ear reference electrode; and A2 = right ear reference electrode), according to the 10-20 standard international position system.19 Monaural clicks at 19.1 clicks per second and a 70dB intensity were used. Test scanning was 2000 clicks with a 15.1 millisecond recording window. A 100Hz low-pass filter and a 3000Hz high-pass filter were used. Stimuli were issued through ER3-A insert earphones and responses were recorded twice for reliability.

For MLAEP recording, electrodes were placed on the brow (Fpz = ground electrode), on the temporal lobes (C3 = left temporal lobe active electrode and C4 = right temporal lobe active electrode), and ear lobes (A1 = left ear reference electrode and A2 = right ear reference electrode), according to the 10-20 standard international position system.19 Monaural clicks at 9.9 clicks per second and a 70dB intensity were used. Test scanning was 1000 clicks with a 99.8 millisecond recording window. A 30Hz to 100Hz filter was used. Stimuli were issued through ER3-A insert earphones and responses were recorded twice for reliability.

For P300 recording, surface electrodes were placed on the brow (Fpz = ground electrode), on the cranial vertex (Cz = active electrode), ear lobules (A1 = left ear reference electrode; and A2 = right ear reference electrode), according to the 10-20 standard international position system.^19^ Instructions were then given about the evaluation. Patients were asked to mentally count only the rare sounds within a series of frequent sounds they would hear; rare sounds did not appear sequentially. Before recording, we trained the patients to make sure they had understood the test procedure.

Binaural stimulation was issued through ER3-A insert earphones, using a 70dB intensity tone burst at 1000Hz as the frequent stimulus (80% probability) and at 2000Hz as the rare stimulus (20% probability). There were 1.1 stimuli per second, and a 1 to 100Hz filter was used. There were 300 frequent stimuli and the recording window was 512 milliseconds.

### Criteria for result analysis

The statistical analysis of AEPs was both quantitative and qualitative. Absolute latency values and interpeak intervals (BAEP), Pa wave and Na-Pa amplitude latency values (MLAEP) and latency values and amplitude (P300) were used for the quantitative analysis. In the qualitative analysis, results were classified as normal or altered, and the types of alterations were described.

BAEP results were classified as normal or altered for each subject according to the wave I, III and V absolute latency values and the interpeak intervals I-III, III-V and I-V for subjects over 24 months, as proposed by Hood^20^ (Frame 1).

**Frame 1:** Normalcy patterns for wave I, III and V absolute latency values and interpeak intervals I-III, III-V and I-V for subjects over 24 months (Hood20).

	Wave I	Wave III	Wave V	Interpeak I-III	Interpeak III-V	Interpeak I-V	
Mean	1,6	3,7	5,6	2,0	1,8	3,8	ms
Standard deviation	0,2	0,2	0,2	0,4	0,4	0,4	ms

Next, the types of alterations seen in each test were described. Auditory pathway changes may be low brainstem alterations (when wave III and V latency values and consequently interpeaks I-III and I-V are increased), high brainstem alterations (when wave V latency values and interpeaks III-V and I-V are increased), or both (when both changes occur simultaneously in the same subjects).

Pa wave latency values in MLAEP were studied only quantitatively. Na-Pa amplitude values were studied both qualitatively and quantitatively.

A difference over 50% between Pa wave amplitudes obtained by comparing the ipsilateral and contralateral modes (C3/A1, C4/A2, C3/A2, C4/A1) two by two is used to indicate dysfunction. Dysfunction may be seen in the electrode effect, which is the difference obtained when comparing Pa wave amplitude measurements with the electrodes placed over each hemisphere (comparisons between C3/A1 and C4/A1; and between C3/A2 and C4/A2). The ear effect occurs when one of the ears, regardless of the electrode site (comparison between C3/A1 and C3/A2; and between C4/A1 and C4/A2) shows constantly decreased Pa wave amplitudes.^21^

Thus, MLAEPs were initially classified as normal or altered, after which the types of alterations were described: ear effect, electrode effect or both (when the same subject has both types of alterations).

Latency values and P300 wave amplitude values were used in the P300 analysis; amplitude values were used only in the quantitative analysis.

The non-target stimulus waveforms were subtracted from the target stimulus waveforms to identify the P300.^21^ Next, latencies were marked on the highest peak - the point of maximum wave amplitude - and amplitudes were measured from the wavepeak to the baseline.^22^

Values were within normal limits in the latency qualitative analysis for the age range we investigated (between 225 and 365 milliseconds for ages 17 to 30 years, and between 290 and 380 milliseconds for ages 30 to 50 years).^16^ Results were considered as altered when latency values were increased or when responses were absent. After P300 was classified as normal or altered, the types of alterations were described as: increased latency, absent responses or both (increased latency and absent responses occurring simultaneously in the same subject).

### Statistical Analysis

The Friedman, Wilcoxon, Mann-Whitney and Equality of Two Proportions non-parametric tests were used in this study. The confidence interval for the mean was also used in the descriptive analysis.

The result of each comparison has a p-value, which provides a conclusion about the test.

The confidence interval for the mean is used when verifying by how much the mean may vary within a certain confidence probability.

The significance level was 0.05 (5%) and the statistical confidence interval was 95%.

## RESULTS

This part was divided into three to better explain the results.

### Part 1: BAEP

Quantitative and qualitative BAEP results for the study and control groups are presented below.

### Quantitative Analysis

Table 1 shows the quantitative analysis of BAEP results for both groups.

**Table 1:** Comparison of absolute latencies I, III, V and interpeak intervals I-III, III-V, I-V between the control and study groups.

BAEP			Mean	Median	Standard Deviation	Quartile 1	Quartile 3	Size	CI	p-valor
RE	I	CG	1,62	1,60	0,10	1,53	1,70	30	0,03	0,103
		SG	1,67	1,70	0,12	1,60	1,80	30	0,04	
	III	CG	3,65	3,60	0,13	3,53	3,70	30	0,05	0,046*
		SG	3,73	3,70	0,16	3,60	3,80	30	0,06	
	V	CG	5,60	5,60	0,11	5,50	5,70	30	0,04	0,175
		SG	5,65	5,60	0,17	5,50	5,80	30	0,06	
	I-III	CG	2,03	2,00	0,07	2,00	2,10	30	0,03	0,202
		SG	2,06	2,10	0,14	2,00	2,10	30	0,05	
	III-V	CG	1,95	1,90	0,11	1,90	2,00	30	0,04	0,264
		SG	1,92	1,90	0,11	1,90	2,00	30	0,04	
	I-V	CG	3,97	4,00	0,10	3,90	4,00	30	0,04	0,702
		SG	3,98	4,00	0,15	3,90	4,10	30	0,06	
LE	I	CG	1,67	1,70	0,10	1,60	1,70	30	0,03	0,810
		SG	1,67	1,70	0,10	1,60	1,70	30	0,04	
	III	CG	3,71	3,70	0,15	3,60	3,80	30	0,05	0,952
		SG	3,72	3,70	0,14	3,60	3,80	30	0,05	
	V	CG	5,66	5,60	0,15	5,60	5,70	30	0,06	0,715
		SG	5,66	5,65	0,14	5,60	5,70	30	0,05	
	I-III	CG	2,05	2,05	0,10	2,00	2,10	30	0,04	0,994
		SG	2,05	2,00	0,12	2,00	2,10	30	0,04	
	III-V	CG	1,95	1,90	0,10	1,90	2,00	30	0,04	0,783
		SG	1,94	1,95	0,12	1,83	2,00	30	0,04	
	I-V	CG	3,99	4,00	0,13	3,90	4,00	30	0,05	0,615
		SG	3,99	4,00	0,15	3,90	4,08	30	0,05	

CG Control group SG Study group RE Right ear LE Left ear

### Qualitative Analysis

Tables 2 and 3 show the qualitative analysis of BAEP results in the study and control groups.

**Table 2:** Distribution of normal and altered BAEP results in the control and study groups.

BAEP	CG		SG		p-valor
	Qtty	%	Qtty	%	
Normal	28	93,3%	28	93,3%	1,000
Altered	2	6,7%	2	6,7%	
p-valor	<0,001*		<0,001*		

CG Control group SG Study group QTTY Quantity

**Table 3:** Distribution of the types of alterations found in BAEP in the control and study groups.

BAEP	Types of alterations					
	Low brainstem		High brainstem		Both	
	Qtty	%	Qtty	%	Qtty	%

CG	2	100,0%	0	0,0%	0	0,0%
SG	2	100,0%	0	0,0%	0	0,0%

p-valor	- x -		- x -		- x -	

CG Control group SG Study group QTTY Quantity

### Part 2: MLAEP

Quantitative and qualitative MLAEP results for the study and control groups are presented below.

### Quantitative Analysis

Tables 4 and 5 show the quantitative analysis of MLAEP results in the study and control groups.

**Table 4:** Comparison of MLAEP Pa wave latencies for C3/A2, C4/A2, C3/A1 and C4/A1 between the control and study groups.

MLAEP	RE				LE			
	C3/A2		C4/A2		C3/A1		C4/A1	
	CG	SG	CG	SG	CG	SG	CG	SG
Mean	32,03	30,74	32,14	31,16	32,73	30,76	32,05	31,82
Median	31,59	31,57	31,69	31,57	32,74	30,69	31,98	32,16
Standard deviation	3,18	4,13	3,15	4,16	3,44	4,74	3,39	5,52
Quartile 1	30,42	27,77	30,41	27,87	30,85	28,26	30,42	29,18
Quartile 3	33,81	33,13	33,44	34,69	34,30	34,59	33,82	35,08
Size	30	30	30	30	30	30	30	30
CI	1,14	1,48	1,13	1,49	1,23	1,69	1,21	1,98

p-valor	0,264		0,544		0,081#		0,739	

CG Control group SG Study group RE Right ear LE Left ear

**Table 5:** Comparison of MLAEP Na-Pa amplitudes for C3/A2, C4/A2, C3/A1 and C4/A1 between the control and study groups.

MLAEP	RE				LE			
	C3/A2		C4/A2		C3/A1		C4/A1	
	CG	SG	CG	SG	CG	SG	CG	SG
Mean	1,29	2,34	1,22	1,69	1,21	1,54	1,30	1,93
Median	1,30	1,80	1,05	1,49	1,10	1,13	1,09	1,29
Standard deviation	0,64	2,44	0,66	1,42	0,62	1,87	0,67	2,33
Quartile 1	0,76	0,90	0,78	0,76	0,74	0,83	0,89	0,71
Quartile 3	1,55	2,86	1,46	2,09	1,66	1,76	1,64	1,92
Size	30	30	30	30	30	30	30	30
CI	0,23	0,87	0,24	0,51	0,22	0,67	0,24	0,84

p-valor	0,099#		0,209		0,796		0,706	

CG Control group SG Study group RE Right ear LE Left ear

### Qualitative Analysis

Tables 6 and 7 show the qualitative analysis of MLAEP results in the study and control groups.

**Table 6:** Distribution of normal and altered MLAEP results in the control and study groups.

MLAEP	CG		SG		p-valor
	Qtty	%	Qtty	%	
Normal	20	66,7%	15	50,0%	0,190
Altered	10	33,3%	15	50,0%	
p-valor	0,010*		1,000		

CG Control group SG Study group QTTY Quantity

**Table 7:** Distribution of the types of alterations found in MLAEP in the control and study group subjects.

MLAEP	Types of alterations					
	Ear effect		Electrode effect		Both	
	Qtty	%	Qtty	%	Qtty	%
CG	5	50,0%	1	10,0%	4	40,0%
SG	4	26,7%	0	0,0%	11	73,3%

p-valor	0,234		0,211		0,096#	

CG CG - Control group SG Study group QTTY Quantity

### Part 3: Long-latency AEPs (P300)

In this last segment, quantitative and qualitative P300 results for the study and control groups are presented.

### Quantitative Analysis

Tables 8 and 9 show the quantitative analysis of P300 results in the study and control groups.

**Table 8:** Comparison of P300 latencies between the control and study groups.

P300	RE		LE	
	CG	SG	CG	SG
Mean	307,99	312,35	308,77	308,74
Median	306,20	313,20	306,20	300,20
Standard deviation	39,28	35,51	37,26	33,28
Quartile 1	287,20	289,70	286,20	285,20
Quartile 3	334,70	330,20	330,20	325,20
Size	28	26	28	26
CI	14,55	13,65	13,80	12,79

p-valor	0,762		0,890	

CG Control group SG Study group RE Right ear LE Left ear

**Table 9:** Comparison of P300 amplitudes between the control and study groups.

P300	RE		LE	
	CG	SG	CG	SG
Mean	6,66	7,96	6,67	7,53
Median	5,45	6,77	5,80	7,05
Standard deviation	3,56	4,99	3,47	4,06
Quartile 1	4,67	5,25	4,69	5,30
Quartile 3	8,30	9,50	8,13	8,44
Size	28	26	28	26
CI	1,32	1,92	1,29	1,56

p-valor	0,180		0,203	

CG Control group SG Study group RE Right ear LE Left ear

### Qualitative Analysis

Tables 10 and 11 show the qualitative analysis of P300 results in the study and control groups.

**Table 10:** Distribution of normal and altered P300 results in the control and study groups.

P300	CG		SG		p-valor
	Qtty	%	Qtty	%	
Normal	27	90,0%	25	83,3%	0,448
Altered	3	10,0%	5	16,7%	

p-valor	<0,001*		<0,001*		

CG Control group SG Study group Qtty Quantity

**Table 11:** Distribution of the types of alterations found in P300 in the control and study groups.

P300	Types of alterations					
	Increased latency		No response		Both	
	Qtty	%	Qtty	%	Qtty	%
CG	1	33,3%	2	66,7%	0	0,0%
SG	1	20,0%	4	80,0%	0	0,0%

p-valor	0,673		0,211		-x-	

CG Control group SG Study group Qtty Quantity

## DISCUSSION

We noted before analyzing the results that during our survey we found few papers on SLE patients, especially studies on AEPs in SLE.

We studied central auditory function in SLE patients by means of the AEPs.

In clinical audiology, the frequency of associating objective auditory assessment methods with subjective behavioral methods has increased, which has raised the diagnostic precision of central and/or cognitive auditory disorders. Objective methods currently used by health professionals include the investigation of AEPs to assess the neuroelectrical activity of auditory pathways from the auditory nerve to the cerebral cortex in response to an acoustic stimulus or event.^7^

### Part 1: BAEP

Quantitative and qualitative statistical analyses were made of the BAEP in each group.

Absolute waves I, III, V latencies and interpeak intervals I-III, III-V, I-V were initially compared among the study and control groups in the quantitative statistical analysis; there was only a statistically significant difference in the right ear wave III (Table 1).

The mean BAEP results in this study for the study and control group is in accordance with the normalcy standards suggested by Hood.^20^ This author suggests the following values for individuals over 24 months: Wave I: 1.6ms (standard deviation ± 0.2ms), Wave III: 3.7ms (standard deviation ± 0.2ms), Wave V: 5.6ms (standard deviation ± 0.2ms), Interpeak I-III: 2.0ms (standard deviation ± 0.4ms), Interpeak III-V: 1.8ms (standard deviation ± 0.4ms), Interpeak I-V: 3.8ms (standard deviation ± 0.4ms).

Results in the qualitative statistical analysis were initially classified as normal or altered when comparing the study and control groups (Table 2), after which the types of alterations were classified (Table 3). In comparing normal and altered results, both groups had the same number of normal results (93.3%) and altered results (6.7%); thus, there were no statistically significant differences between both groups. The only alteration that was seen in both groups was found in the low brainstem results.

These findings suggest that brainstem auditory pathways in SLE subjects function similarly to those of individuals in the control group.

Our findings are similar to those of Ávila,^23^ who evaluated BAEP in 33 SLE patients and found no alterations.

Mongey et al.^24^ undertook neurophysiologic studies of SLE subjects and found altered BAEP in six individuals.

Costallat et al. ^25^ also found altered BAEP in an SLE patient.

We also found altered BAEP in SLE subjects, but altered results in the study group were not statistically significant compared to the control group.

### Part 2: Mid-latency AEPs

Quantitative and qualitative statistical analyses of MLAEP between both groups were undertaken. MLAEP Na-Pa latencies and amplitudes in C3/A2, C4/A2, C3/A1 and C4/A1 modes were compared between the study and control groups (Tables 4 and 5) for the quantitative statistical analysis. In the qualitative statistical analysis, results were initially classified as normal and altered when comparing both groups (Table 6), after which the types of alterations were classified as the ear effect, the electrode effect, or both (Table 7).

The quantitative statistical analysis of MLAEP revealed no statistically significant differences in Pa wave latencies or in Na-Pa amplitudes in C3/A2, C4/A2, C3/A1 and C4/A1 modes between the study and control groups (Tables 4 and 5).

Latency values in our results are in accordance with Schochat's^14^ normal standard values, which show that the Pa wave occurs about 30ms after the acoustic stimulus. There are no established normal amplitude values, given the wide intersubject variability; this type of analysis should be a comparative intrasubject observation between hemispheres. The amplitude value of one hemisphere, regardless of reference electrode placement, should not be higher than 50% of the opposite hemisphere.^7^

Costa et al.26 studied the MLAEP of 77 children and found that the Na wave latency occurred about 15.02ms to 29.23ms, and that the Pa wave latency occurred about 23.38 to 35.25ms. The Na-Pa amplitude was between 0.4 and 2.58 µV. These authors concluded that there is ample intersubject variability in latencies and amplitudes.

In the qualitative statistical analysis, results were initially classified as normal and altered in the comparison between both groups (Table 6), after which the types of alterations seen were classified (Table 7). In this comparison, we found that the percentage of normal results in the control group was 66.7% (and therefore, 33.3% of altered results). In the study group, the percentage of normal results was 50% (and 50% of altered results). The difference between both groups, however, is not statistically significant. In the control group, we found ear effect alterations in 50%, the electrode effect in 10% and both in 40% of the altered results. In the study group, we found ear effect alterations in 26.7% and both effect in 73.3% of the altered results.

We also found a high rate of altered MLAEP results in both the study and control groups. As a result of these findings, we believe that we should have undertaken a behavioral assessment of auditory processing in our sample subjects to discard auditory processing alterations that might explain such changes.

Bruner^27^ studied auditory processing in SLE subjects by using behavioral tests and found that SLE subjects performed worse that controls, especially when there were neuropsychiatric conditions.

### Part 3: Long-latency AEPs

This study included quantitative and qualitative statistical analyses of long-latency AEPs (P300). In the quantitative statistical analysis, P300 latency values and amplitudes between both groups were compared (Tables 8 and 9). In the qualitative statistical analysis, results were initially classified as normal and altered in the comparison between both groups (Table 10), after which the types of alterations - increased latency, absent responses or both - were classified (Table 11).

There were no statistically significant differences in the quantitative statistical analysis of P300 latencies and amplitudes between the study and control groups (Tables 8 and 9).

There were no statistically significant differences in the qualitative statistical analysis of normal and altered results between the study and control groups (Table 10). In the control group, increased latency was responsible for 33.3% of altered results; 66,7% of the control group had absent responses. Increased latency was seen in 20% of the study group; absent responses were present in 80% of the altered results in the study group (Table 11).

Our findings are within the normal standards of this potential, as suggested by Musiek and Berge,28 (latency values around 250 to 500ms and amplitude ranging from 7 to 25µV.

Colafemina et al.^29^ assessed the auditory P300 in 20 healthy normal hearing young adults of both genders aged between 21 and 35 years and found similar results to our findings. Mean P300 latency and amplitude values were 310.92ms and 4.32µV.

Our findings are different from those in Ito et al.'s^30^ paper. These authors evaluated long-latency AEPs in 17 SLE patients and found that N100 and P200 latencies were within normal limits in all patients, while P300 latencies were significantly prolonged regardless of the presence or absence of cognitive dysfunction.

Avila^23^ assessed P300 values in 33 SLE patients and found no changes in this potential.

The P300 has been widely used in medical practice, especially for assessing cognitive function, attention and memory.^15^, ^31^, ^32^ Due to this, we believed that SLE subjects would have worse results compared to the control group, given that Sabbadini et al.^3^ had found compromised short and long term memory processing, and decreased verbal and visual information processing in these subjects with or with no neuropsychiatric conditions. These authors also found that attention may be significantly compromised, especially in SLE patients with neuropsychiatric manifestations.

We did not know whether the subjects in the study group had neuropsychiatric involvement, since this information was not available in their charts. Faced with our P300 results in the study group, we believe that these subjects had no neuropsychiatric conditions.

## CONCLUSION

A critical analysis of the data obtained by testing short, mid and long latency AEPs in LES individuals suggests that there is no difference between the BAEP, MLAEP and long latency AEPs (P300) of the study and control group subjects.

## References

[1] Sato EI (1998). Doenças Reumáticas Auto-imunes. Diagn Tratamento.

[2] Appenzeller S, Costallat L, Lavras T (2003). Comprometimento primário do sistema nervoso central no lúpus eritematoso sistêmico. Rev Bras Reumatol.

[3] Sabbadini MG, Manfredi AA, Bozzolo E, Ferrario L, Rugarli C, Scorza R (1999). Central nervous system involvement in systemic lupus erythematosus patients without overt neuropsychiatric manifestations. Lúpus.

[4] Andonopoulus AP, Naxakis S, Goumas P, Lygatsikas C (1995). Sensorineral hearing disorders in systemic lupus erythematosus: A controlled study. Clin Exp Rheumatol.

[5] Kastanioudakis I, Ziavra N, Voulgari PV, Exarchakos G, Skevas A, Drosos AA (2002). Ear involvement in systemic lupus erythematosus patients: a comparative study. J Laryngol Otol.

[6] Cecatto SB, Garcia RID, Costa KS, Anti SMA, Longone E, Rapoport PB (2004). Perda auditiva sensorioneural no lúpus eritematoso sistêmico: relato de três casos. Rev Bras Otorrinolaringol.

[7] Junqueira CAO, Frizzo ACF, Aquino AMCM (2002). Processamento Auditivo: Eletrofisiologia e Psicoacústica.

[8] Matas CG, Frazza MM, Munhoz MSL (1998). Neonatologia: Um convite à atuação fonoaudiológica.

[9] Musiek FE, Gollegly KM, Bess FH (1988). Hearing Impairment in Children.

[10] Matas CG, Carvallo RMM (2003). Fonoaudiologia Informação para a Formação: Procedimentos em Audiologia.

[11] Möller AR, Jannetta P, Bennett M, Möller MB (1981). Intracranially recorded responses from human auditory nerve: new insights into the origin of brainstem evoked potentials. Electroencephalogr and Clin Neurophysiol.

[12] Ribeiro FM (2001). Atuação fonoaudiológica no ambiente hospitalar.

[13] Ganança MM, Munhoz MSL, Caovilla HH, Silva MLG (2004). Condutas na vertigem.

[14] Schochat E, Carvallo RMM (2003). Fonoaudiologia Informação para a Formação: Procedimentos em Audiologia.

[15] Schochat E, Carvallo RMM (2003). Fonoaudiologia Informação para a Formação: Procedimentos em Audiologia.

[16] Mc Pherson DL (1996). Late Potentials of the Auditory System.

[17] Tan EM, Cohen AS, Fries JF, Mais AT, Mc Shane DJ, Rothfield NF (1982). The 1982 revised criteria for the classification of systemic lupus erythematosus. Arthritis Rheum.

[19] Jasper HH (1958). Report of the committee on methods of clinical examination in electroencephalography. Eletroenceph Clin Neurophysiol.

[20] Hood LJ (1998). Clinical applications of the auditory brainstem response.

[21] Musiek FE, Lee WW, Musiek FE, Rintelmann WF (2001). Perspectivas atuais em avaliação auditiva.

[22] Junqueira CAO, Colafêmina JF (2002). Investigação da estabilidade inter e intra-examinador na identificação do P300 auditivo: análise de erros. Rev Bras Otorrinolaringol.

[23] Ávila JO (1998). O emprego do potencial de longa latência P300(P3) no diagnóstico de complicações neurológicas e psíquicas no Lúpus Eritematoso Sistêmico; comparação com a avaliação clínica e neuropsicológica [tese].

[24] Mongey AB, Glynn D, Hutchinson Mayor, Bresnihan B (1987). Clinical neurophysiology in the assessment of neurological symptoms in systemic lupus erythematosus. Rheumatol Int.

[25] Costallat LT, Quagliato EM, Zanardi VA (1997). Evoked potentials in the assessment of neuropsychiatric manifestations in systemic lupus erythematosus. Clin Rheumatol.

[27] Bruner AP (2006). Avaliação do Processamento Auditivo em pessoas com Lúpus Eritematoso Sistêmico: Escuta dicótica e processamento temporal [tese].

[29] Colafêmina JF, Fellipe ACN, Junqueira AO, Frizzo ACF (2000). Potenciais evocados auditivos de longa latência (P300) em adultos jovens saudáveis: um estudo normativo. Rev Bras Otorrinolaringol.

[30] Ito J, Suwazono S, Kimura J, Shibasaki H (1993). Auditory event-related potentials in patients with systemic lupus erythematosus. Eur Neurol.

[31] Desmedt JE, Debecker J (1979). Slow potential shifts and decision P350 interactions in tasks with random sequences of near-threshold clicks and finger stimuli delivered at regular intervals. Eletroenceph Clin Neurophysiol.

[32] Goodin DS (1990). Clinical utility of long latency cognitive event- related potentials (P3): the pros. Eletroenceph Clin Neurophysiol.

